# Predicting the risk factors of diabetic ketoacidosis-associated acute kidney injury: A machine learning approach using XGBoost

**DOI:** 10.3389/fpubh.2023.1087297

**Published:** 2023-04-06

**Authors:** Tingting Fan, Jiaxin Wang, Luyao Li, Jing Kang, Wenrui Wang, Chuan Zhang

**Affiliations:** ^1^Department of Endocrinology, Second Affiliated Hospital of Jilin University, Changchun, China; ^2^Digestive Diseases Center, Department of Hepatopancreatobiliary Medicine, Second Affiliated Hospital of Jilin University, Changchun, China

**Keywords:** diabetic ketosis, acute kidney injury, machine learning, XGBoost, outcome

## Abstract

**Objective:**

The purpose of this study was to develop and validate a predictive model based on a machine learning (ML) approach to identify patients with DKA at increased risk of AKI within 1 week of hospitalization in the intensive care unit (ICU).

**Methods:**

Patients diagnosed with DKA from the Medical Information Mart for Intensive Care IV (MIMIC-IV) database according to the International Classification of Diseases (ICD)-9/10 code were included. The patient’s medical history is extracted, along with data on their demographics, vital signs, clinical characteristics, laboratory results, and therapeutic measures. The best-performing model is chosen by contrasting the 8 Ml models. The area under the receiver operating characteristic curve (AUC), sensitivity, accuracy, and specificity were calculated to select the best-performing ML model.

**Results:**

The final study enrolled 1,322 patients with DKA in total, randomly split into training (1,124, 85%) and validation sets (198, 15%). 497 (37.5%) of them experienced AKI within a week of being admitted to the ICU. The eXtreme Gradient Boosting (XGBoost) model performed best of the 8 Ml models, and the AUC of the training and validation sets were 0.835 and 0.800, respectively. According to the *result of feature importance*, the top 5 main features contributing to the XGBoost model were blood urea nitrogen (BUN), urine output, weight, age, and platelet count (PLT).

**Conclusion:**

An ML-based individual prediction model for DKA-associated AKI (DKA-AKI) was developed and validated. The model performs robustly, identifies high-risk patients early, can assist in clinical decision-making, and can improve the prognosis of DKA patients to some extent.

## 1. Introduction

Diabetic ketoacidosis (DKA) is a serious acute complication of diabetes mellitus that can be fatal if left untreated. DKA is characterized by uncontrolled blood glucose (BG) levels, acidosis, and ketosis. It can also cause imbalances in electrolytes and fluids, leading to complications such as cerebral edema, acute kidney injury (AKI), and even renal failure in severe cases (1, 2). AKI is a common complication, affecting 40 to 50 percent of DKA patients. Unfortunately, AKI can result in increased morbidity and mortality, prolonged intensive care unit (ICU) stays, a greater susceptibility to chronic kidney disease (CKD), and recurrent AKI episodes during ICU treatment. Therefore, it is critical to closely monitor DKA patients for signs of AKI and promptly intervene to mitigate its potential negative impact (3, 4). The diagnosis of AKI is typically based on the dynamic changes in serum creatinine (SCr) and urine output, following the clinical practice recommendations established by the Kidney Disease Improving Global Outcomes (KDIGO) organization (5). However, renal damage usually precedes the elevation of SCr levels, so that renal damage has already begun by the time AKI is diagnosed (6). DKA-associated AKI (DKA-AKI) usually occurs after hypoperfusion of the kidney due to hypovolemia. Renal function can be improved to some extent by effective prevention and treatment, such as applying vasoactive drugs, ensuring adequate renal perfusion, and avoiding nephrotoxic drugs (7). Consequently, it is necessary to explore predictors for AKI and monitor the population at risk for DKA-AKI. Clinicians can improve the prognosis of DKA patients with timely management.

Several studies have demonstrated that various factors, including age, type of diabetes, comorbidities, respiratory rate (RR), blood pressure, baseline Scr, blood urea nitrogen (BUN), and urine output, can be used to predict the risk of DKA-AKI (3, 4, 8). In our previous work (9), a model for predicting DKA-AKI risk based on logistic regression was developed and a nomogram was drawn. Besides, numerous models for predicting AKI have been developed (10–13). However, few publications have identified the specific risk of AKI in DKA patients.

In recent years, machine learning (ML) algorithms have been found to have excellent predictive performance (14). ML has also demonstrated excellent performance in the administration of ICU patients, and combining it with electronic health record systems can increase the reliability of technological support for critical care.

In the administration of ICU patients, ML has demonstrated excellent performance and, when combined with electronic health record systems, can increase the reliability of technological support for critical care (15). Unfortunately, to our knowledge, there are no relevant studies that apply machine learning algorithms to build a model and identify risk factors for DKA-AKI. Therefore, in this research, we developed a model for predicting DKA-AKI in real-time using ML algorithms and validated its performance. This is an important step toward identifying and managing DKA-AKI, as it could help clinicians intervene early and prevent further complications.

## 2. Methods

### 2.1. Database

Patients’ information for this retrospective investigation was obtained from the database called Marketplace for Medical Information in Intensive Care IV (MIMIC-IV). It is consisted of the medical history for the ill critical patients at Beth Israel Deaconess Medical Center (Boston, MA), including demographic information, disease diagnosis, vital signs, laboratory tests, treatment information, survival status, and other comprehensive clinical records. The inclusion of the MIMIC-IV database has been expanded from 2008–2012 to 2008–2019, compared to the previous version of the MIMIC-III (Certificate number: 9168028).

### 2.2. Study population

**Figure 1:**
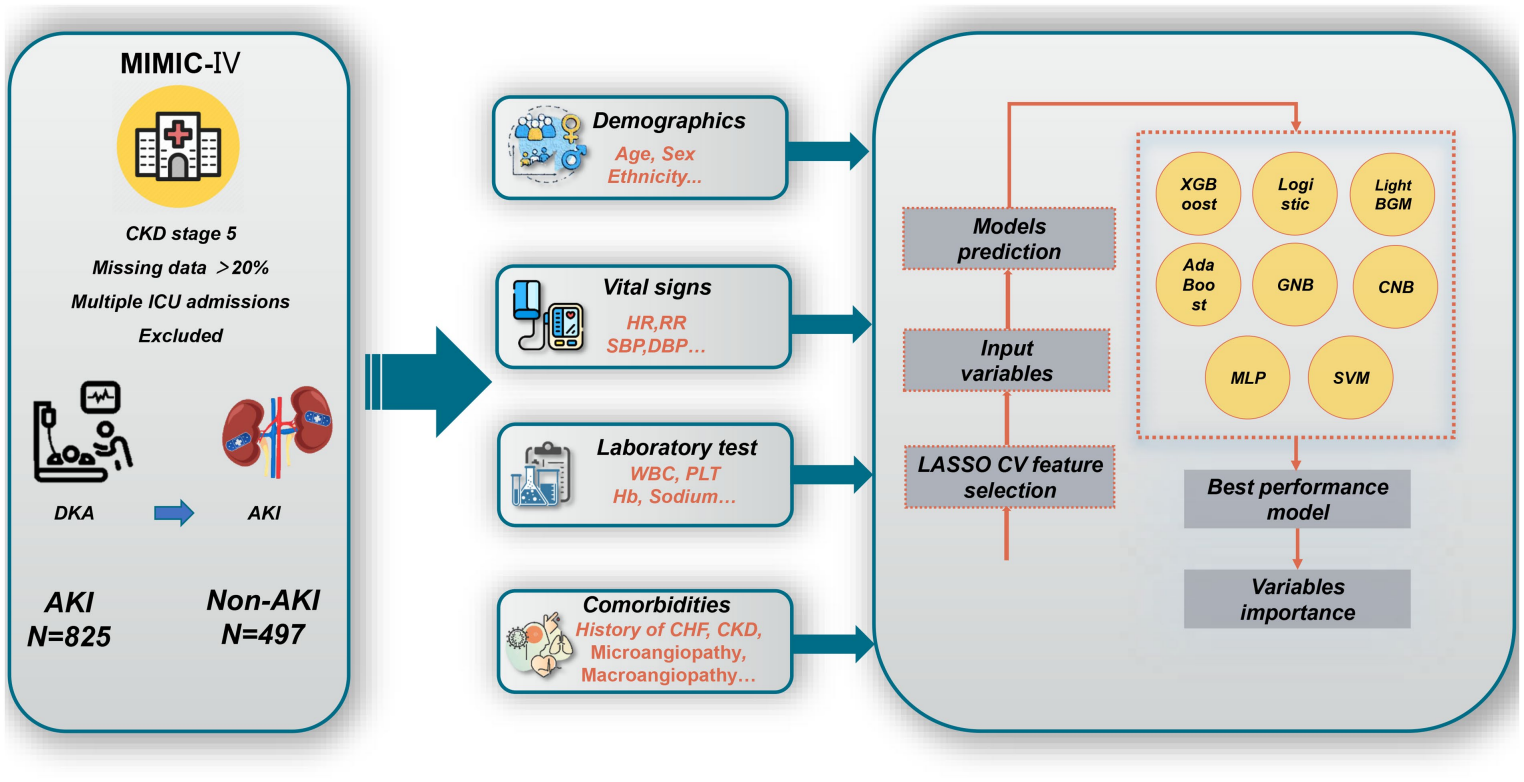
Overall flowchart of this study. MIMIC-IV, Medical Information Mart for Intensive Care IV; CKD, chronic kidney disease; ICU, intensive care unit; DKA, v; AKI, acute kidney injury; HR, heart rate; RR, respiratory rate; WBC, white blood cell count; PLT, Platelet count; Hb, hemoglobin; CHF, congestive heart failure; XGBoost, eXtreme Gradient Boosting; GNB, Gaussian Naïve Bayes; CNB, Complement Naive Bayes; MLP, multi-layer perceptron neural network; SVM, support vector machine; LASSO CV, least absolute shrinkage and selection operator cross-validation.

DKA patients in the MIMIC-IV databases identified with the International Classification of Diseases (ICD) -9/10 code were included. The excluded criteria were: (1) patients with a diagnosis of CKD stage 5; (2) if a patient was admitted to the ICU repeatedly during one hospitalization, only the first hospitalization information was retained; and (3) patients with more than 20% missing data. The overall flowchart is shown in Figure 1.

### 2.3. Data extraction and pre-processing

The following patient data were retrieved from the MIMIC-IV database: demographics, vital signs, comorbidities, laboratory tests, interventions, prognosis, and scoring systems, as shown in Supplementary Table S1. Features missing greater than 20% were excluded in the follow-up study, and other variables were duplicated using nearest neighbor imputation algorithms (excluded missing variables were shown in Supplementary Table S2). All items were recorded within 24 h of admission to the ICU. And due to repeat sampling, we only retained the results of the first test.

### 2.4. Outcome

Our study’s primary outcome was the occurrence of AKI of DKA patients receiving ICU care within a week. Utilizing KDIGO criteria, a diagnosis of AKI was determined (5).

### 2.5. Model development and validation

Supervised ML algorithms have been playing an important role in various clinical prediction models. In the present study, supervised ML algorithms were used to construct later predictions of the risk of AKI in DKA patients. The least absolute shrinkage and selection operator cross-validation (LASSO CV) method was used for model selection in order to reduce model complexity and the risk of overfitting and to optimize model training speed. Machine learning has contributed massively applications in medical diagnosis, treatment, and prediction (16). In our present study, the overall dataset was divided into 2 groups randomly, 85% in training cohort and 15% in validation cohort, respectively. To minimize overfitting and identify the optimization hyperparameters, 10-fold cross-validation (CV) was also performed. And then, 8 Ml model algorithms, including eXtreme Gradient Boosting (XGBoost); logistic regression, Light BGM, Ada Boost, Gaussian Naïve Bayes (GNB), multi-layer perceptron neural network (MLP), Complement Naive Bayes (CNB), support vector machine (SVM). Before establishing the models, the Lasso CV features selection method was applied to better select feature parameters. Furthermore, we reported several parameters related to model performance in this study, including area under the receiver operating characteristic curve (AUC), sensitivity, specificity, and accuracy. The final model was determined according to the highest AUC value after comparing the performance of the 8 different ML models. Finally, *feature importance* was calculated for the final models to evaluate the contribution of the candidate predictors. The programming written using Python (package version 3.8) was used to exert data analysis.

### 2.6. Statistical analysis

Variables that followed a normal distribution were expressed as the mean ± standard deviation (SD); if they did not follow a normal distribution the quartiles were generally used. Student’s t-test or Mann–Whitney *U*-test was used to analyze continuous variables. In the case of categorical variables, the χ^2^ test or Fisher’s exact test was generally used. All Statistical analyses were performed using R version 3.6.3 and python version 3.7. The core code for model construction was provided by the Extreme Smart Analysis platform^1^ and uploaded to the supplemental material.

## 3. Results

### 3.1. Patients’ characteristics

1,322 patients diagnosed with DKA in total were eventually enrolled in our study. Among the total cohort, 689 (52.1%) were female, and the median age was 50 [IQR 35, 62] years. Within a week of admission to the ICU, 497 (37.6%) progressed to DKA-AKI. The incidence of DKA-AKI was 36.4% (181/497) in stage 1, 34.4% (171/497) in stage 2, and 29.2% (145/497) in stage 3. CRRT was applied to 17.4% (73/497) of AKI patients. The AKI group had higher ventilator utilization, hospital length of stay (HLOS), and in-hospital mortality. Except for gender, systolic blood pressure (SBP), liver disease, history of hypertension, platelet count (PLT), calcium, BG, and infusion volume, the statistical analysis of the candidate predictors revealed significant differences between the AKI and non-AKI groups (Table 1).

**Table 1:** Characteristic at baseline between AKI and non-AKI group.

Variable	Total (*n* = 1,322)	Non-AKI (*n* = 825)	AKI (*n* = 497)	*p* value
Age, years	50 [35, 62]	43 [30, 57]	58 [46, 68]	<0.001
Gender (Female)	689 (52.1)	425 (51.5)	264 (53.1)	0.572
Weight, Kg	73.2 [62.0, 87.1]	70.0 [60.3, 83.6]	78.5 [66.0, 94.0]	<0.001
Ethnicity				0.028
White	742 (56.1)	474 (57.4)	268 (53.9)	
African-American	364 (27.5)	223 (27.0)	141 (28.3)	
Hispanic-American	76 (5.7)	51 (6.2)	25 (5.0)	
Asian	28 (2.1)	21 (2.5)	7 (1.4)	
Other	112 (8.5)	56 (6.8)	56 (11.3)	
DM type				<0.001
T1DM	821 (62.1)	546 (66.2)	275 (55.3)	
T2DM	373 (28.2)	209 (25.3)	164 (33.1)	
Other	128 (9.7)	70 (8.5)	58 (11.7)	
HR, beats/min	100 [88, 111.000]	101 [89, 113]	98 [85, 109]	<0.001
RR, breaths/min	19 [16, 23]	19 [16, 23]	20 [17, 24]	0.002
SBP, mmHg	129 [114, 145]	128 [115, 144]	130 [111, 147]	0.907
DBP, mmHg	71 [60, 83]	72 [62, 83]	69 [56, 82]	0.002
Microangiopathy (Yes)	691 (52.3)	411 (49.8)	280 (56.3)	0.022
Macroangiopathy (Yes)	319 (24.1)	260 (31.5)	59 (11.9)	<0.001
Preexisting CKD				<0.001
Non-CKD	1,006 (76.1)	704 (85.3)	302 (60.8)	
Stage1-3	251 (18.9)	115 (13.9)	136 (27.4)	
Stage3-4	65 (4.9)	6 (0.7)	59 (11.9)	
UTI (Yes)	147 (11.120)	71 (8.606)	76 (15.292)	<0.001
Pneumonia (Yes)	58 (4.387)	18 (2.182)	40 (8.048)	<0.001
Liver disease (Yes)	124 (9.4)	73 (8.8)	51 (10.3)	0.393
History of hypertension (Yes)	513 (38.8)	305 (37.0)	208 (41.9)	0.078
History of CHF (Yes)	208 (15.7)	69 (8.4)	139 (27.9)	<0.001
History of AMI (Yes)	213 (16.1)	83 (10.1)	130 (26.2)	<0.001
History of ACI (Yes)	91 (6.9)	28 (3.4)	63 (12.7)	<0.001
Malignant Cancer (Yes)	59 (4.463)	33 (4.000)	26 (5.231)	0.294
Bicarbonate, mEq/L	21.0 [18.0, 25.0]	21.0 [17.0, 25.0]	22.0 [18.0, 25.0]	0.005
WBC, K/μL	8.2 [5.9, 11.9]	7.8 [5.8, 11.0]	9.0 [6.4, 13.5]	<0.001
PLT, K/μL	228.0 [175.0, 289.0]	227.0 [177.0, 283.0]	229.0 [167.0, 298.0]	0.711
Hb, g/dl	10.9 [9.3, 12.3]	11.4 [10.1, 12.6]	9.8 [8.5, 11.3]	<0.001
Sodium, mEq/L	138.0 [135.0, 140.0]	137.0 [135.0, 140.0]	138.0 [135.0, 141.0]	0.006
Chloride, mEq/L	104.0 [100.0, 108.0]	104.0 [101.0, 108.0]	103.000 [99.0, 109.0]	0.004
Calcium, mEq/L	8.400 [8.0, 8.8]	8.400 [8.0, 8.8]	8.400 [7.9, 8.8]	0.339
Phosphate, mEq/L	2.7 [1.900, 3.700]	2.400 [1.7, 3.2]	3.300 [2.3, 4.5]	<0.001
AG	14.0 [12.0, 17.0]	14.0 [12.0, 17.0]	15.000 [12.0, 18.0]	0.015
Total osmotic pressure	494.600 [440.0, 560.2]	492.0 [437.6, 555.2]	498.400 [447.4, 570.6]	0.024
BUN, mg/dl	16.0 [9.0, 31.0]	13.0 [8.0, 20.0]	28.0 [14.0, 46.0]	<0.001
Scr, mg/dl	0.9 [0.7, 1.5]	0.8 [0.6, 1.1]	1.4 [0.9, 2.5]	<0.001
Potassium, mEq/L	4.1 [3.7, 4.5]	4.0 [3.7, 4.4]	4.2 [3.8, 4.7]	<0.001
Blood glucose, mg/dl	188.0 [135.0, 257.000]	191.0 [139.0, 258.0]	183.000 [130.0, 251.0]	0.174
Infusion volume, mL	12020.0 [7700.0, 17320.000]	11890.0 [7700.0, 16600.0]	12400.000 [7805.0, 18010.0]	0.061
Urine output, mL	1950.0 [1085.0, 3000.0]	2300.0 [1470.0, 3440.0]	1300.000 [600.0, 2250.0]	<0.001
eGFR	0.992 [0.854, 1.093]	1.000 [0.882, 1.114]	0.930 [0.829, 1.067]	<0.001
Use of NaHCO_3_ (Yes)	123 (9.304)	41 (4.970)	82 (16.499)	<0.001
Mechanical ventilation (Yes)	152 (11.498)	31 (3.758)	121 (24.346)	<0.001
GCS	15.000 [15.000, 15.000]	15.000 [15.000, 15.000]	15.000 [15.000, 15.000]	<0.001
CRRT (Yes)	77 (5.825)	4 (0.485)	73 (14.688)	<0.001
OASIS	25.000 [21.000, 31.000]	23.000 [20.000, 27.000]	30.000 [25.000, 39.000]	<0.001
SOFA	3.000 [1.000, 5.000]	2.000 [1.000, 3.000]	5.000 [3.000, 8.000]	<0.001
SAPSII	26.000 [19.000, 36.000]	22.000 [16.000, 29.000]	36.000 [26.000, 45.000]	<0.001
HLOS, days	4.838 [2.969, 8.487]	3.890 [2.647, 6.051]	7.654 [4.158, 13.670]	<0.001
Hospital mortality (Yes)	61 (4.614)	11 (1.333)	50 (10.060)	<0.001

AKI, acute kidney injury; DM, diabetic mellitus; T1DM, type 1 DM; T2DM, type 2 DM; HR, heart rate; RR, respiratory rate; SBP, systolic blood pressure, DBP, diastolic blood pressure, CKD, chronic kidney diseases, UTI, urinary tract infection; CHF, congestive heart failure; AMI, acute myocardial infarction; ACI, acute cerebral infarction; WBC, white blood cell; PLT, platelets count; Hb, hemoglobin; AG, anion gap; BUN, blood urea nitrogen; Scr, serum creatinine; eGFR, estimated glomerular filtration rate; GCS, Glasgow coma scale; OASIS, oxford acute severity of illness score; SOFA, sequential organ failure assessment; SAPS-II, simplified acute physiology score II; HLOS, hospital length of stay.

### 3.2. Predictive model performance

**Figure 2:**
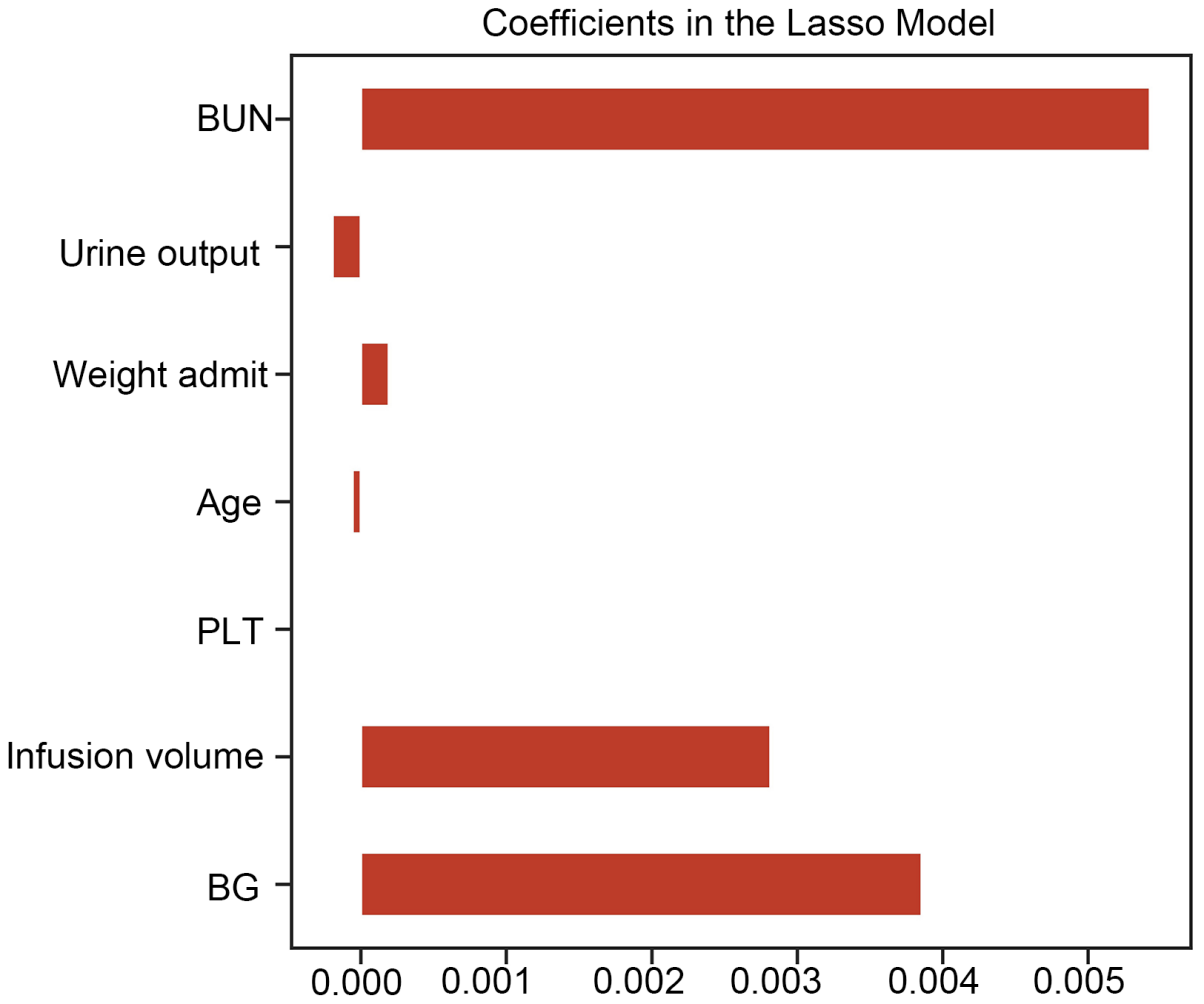
Lasso CV method was used to conduct feature selection. LASSO CV, least absolute shrinkage and selection operator cross-validation, BUN, blood urea nitrogen; BG, blood glucose; PLT, platelet count.

**Figure 3:**
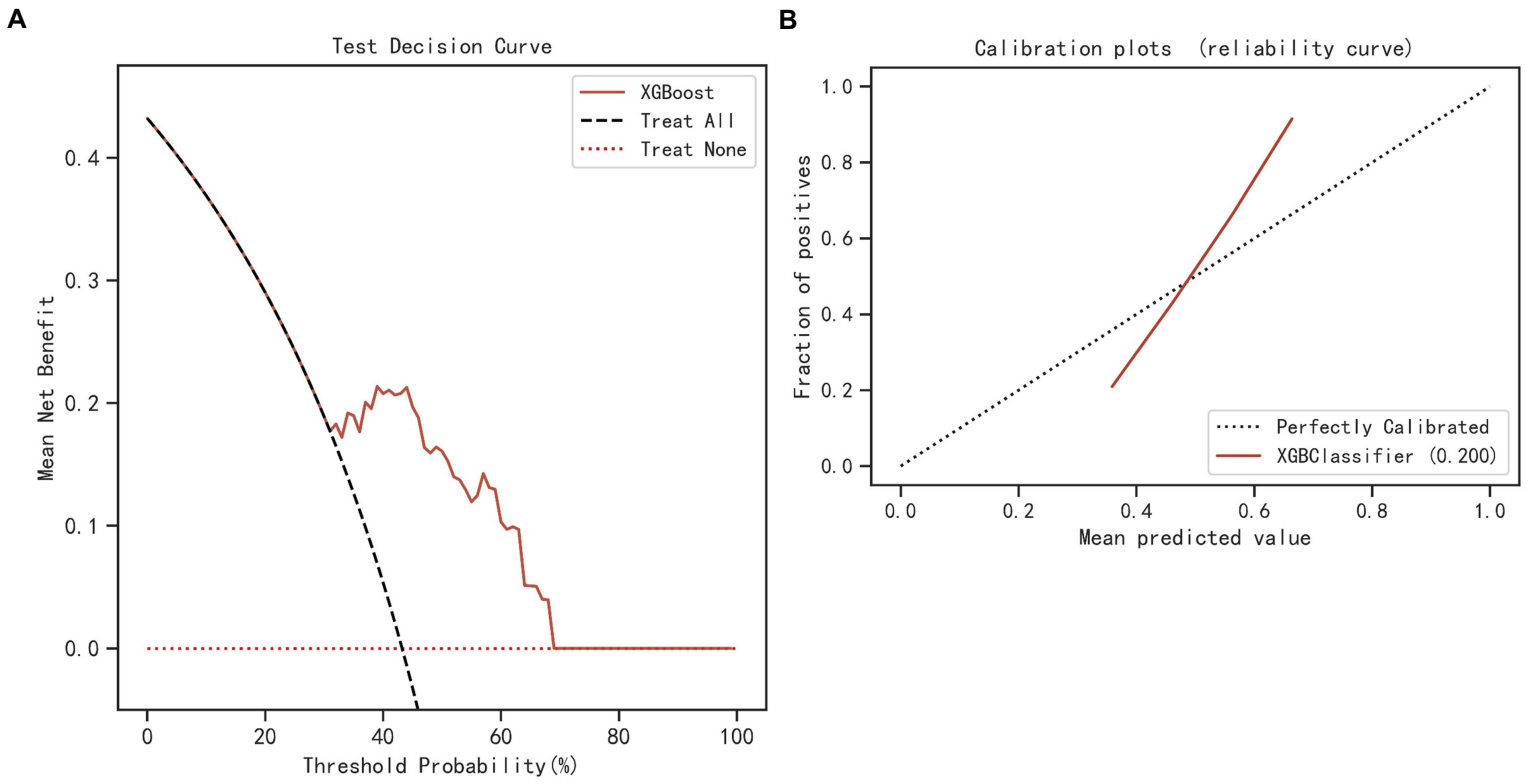
Comparing the different ML models’ AUC in the training **(A)** and validation **(B)** sets. ML, machine learning; AUC, area under the receiver operating characteristic curve; XGBoost, eXtreme Gradient Boosting; GNB, Gaussian Naïve Bayes; CNB, Complement Naive Bayes; MLP, multi-layer perceptron neural network; SVM, support vector machine.

**Figure 4:**
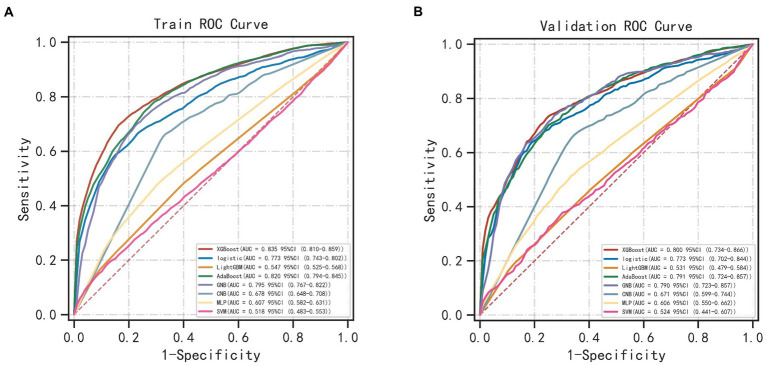
DCA **(A)** and calibration curve **(B)** of the XGBoost and simplified model. DCA, decision curve analysis; XGBoost, eXtreme Gradient Boosting.

The results of LASSO CV screened seven variables as input variables for the model (Figure 2). Eight Ml-based models have been developed and validated, and the XGBoost model performed the strongest in predicting DKA-AKI with the highest AUC value (0.835) (Figure 3A). In addition, the XGBoost model had a sensitivity of 0.716, a specificity of 0.823, and an accuracy of 0.782 in the training set (Table 2). When internal validation was performed, the XGBoost model still showed optimal performance with an AUC of 0.800 (Figure 3B), and its sensitivity, specificity, and accuracy were 0.718, 0.790, and 0.749, respectively (Table 3). Therefore, the XGBoost model was selected as the final mode. Furthermore, decision curve analysis (DCA) (Figure 4A) and calibration plots (Figure 4B) were also performed to further demonstrate the performance of the XGBoost model.

**Table 2:** Model parameters in training set.

Model	AUC	Cutoff	Accuracy	Sensitivity	Specificity	PPV	NPV	F1-Score
XG Boost	0.835 (0.004)	0.463 (0.013)	0.782 (0.007)	0.716 (0.025)	0.823 (0.025)	0.714 (0.022)	0.824 (0.009)	0.714 (0.007)
Logistic	0.773 (0.004)	0.468 (0.033)	0.744 (0.012)	0.627 (0.040)	0.817 (0.042)	0.679 (0.036)	0.782 (0.012)	0.649 (0.007)
Light GBM	0.547 (0.079)	0.600 (0.800)	0.634 (0.031)	0.343 (0.322)	0.777 (0.292)	NA	0.666 (0.052)	NA
AdaBoost	0.820 (0.005)	0.467 (0.002)	0.743 (0.008)	0.770 (0.013)	0.729 (0.019)	0.634 (0.016)	0.837 (0.007)	0.695 (0.006)
GNB	0.795 (0.005)	0.351 (0.032)	0.742 (0.010)	0.720 (0.031)	0.757 (0.031)	0.642 (0.020)	0.817 (0.011)	0.678 (0.010)
CNB	0.678 (0.005)	0.993 (0.022)	0.664 (0.013)	0.660 (0.010)	0.674 (0.007)	NA	0.754 (0.042)	NA
MLP	0.607 (0.074)	0.397 (0.029)	0.612 (0.097)	0.614 (0.282)	0.588 (0.330)	NA	0.710 (0.063)	NA
SVM	0.518 (0.061)	0.449 (0.057)	0.573 (0.101)	0.409 (0.322)	0.673 (0.347)	0.491 (0.084)	0.667 (0.037)	0.363 (0.146)

Data are shown as means ± standard deviations (SD).

AUC, area under the receiver operating characteristic curve; PPV, positive prediction value; NPV, negative prediction value; GNB, Gaussian Naïve Bayes; CNB, Complement Naive Bayes; GNB, Gaussian Naïve Bayes; MLP, multi-layer perceptron neural network; SVM, support vector machine; NA, not applicable.

**Table 3:** Model parameters in validation set.

Model	AUC	Cutoff	Accuracy	Sensitivity	Specificity	PPV	NPV (SD)	F1-Score
XGBoost	0.800 (0.019)	0.463 (0.013)	0.749 (0.017)	0.718 (0.060)	0.790 (0.036)	0.643 (0.035)	0.812 (0.026)	0.677 (0.035)
Logistic	0.773 (0.025)	0.468 (0.033)	0.738 (0.028)	0.650 (0.065)	0.821 (0.064)	0.649 (0.051)	0.788 (0.045)	0.648 (0.050)
Light GBM	0.531 (0.081)	0.600 (0.800)	0.626 (0.042)	0.350 (0.308)	0.751 (0.292)	NA	0.651 (0.038)	NA
AdaBoost	0.791 (0.028)	0.467 (0.002)	0.714 (0.031)	0.695 (0.065)	0.782 (0.053)	0.584 (0.056)	0.820 (0.036)	0.633 (0.052)
GNB	0.790 (0.031)	0.351 (0.032)	0.738 (0.037)	0.694 (0.065)	0.807 (0.073)	0.639 (0.066)	0.809 (0.045)	0.664 (0.057)
CNB	0.671 (0.029)	0.993 (0.022)	0.653 (0.031)	0.677 (0.042)	0.661 (0.028)	NA	0.739 (0.066)	NA
MLP	0.606 (0.077)	0.397 (0.029)	0.617 (0.107)	0.548 (0.305)	0.664 (0.289)	NA	NA	NA
SVM	0.524 (0.093)	0.449 (0.057)	0.569 (0.100)	0.554 (0.292)	0.576 (0.316)	0.467 (0.126)	0.717 (0.146)	0.456 (0.155)

Data are shown as means ± standard deviations (SD).

AUC, area under the receiver operating characteristic curve; PPV, positive prediction value; NPV, negative prediction value; GNB, Gaussian Naïve Bayes; CNB, Complement Naive Bayes; GNB, Gaussian Naïve Bayes; MLP, multi-layer perceptron neural network; SVM, support vector machine; NA, not applicable.

### 3.3. Relative importance of variables

**Figure 5:**
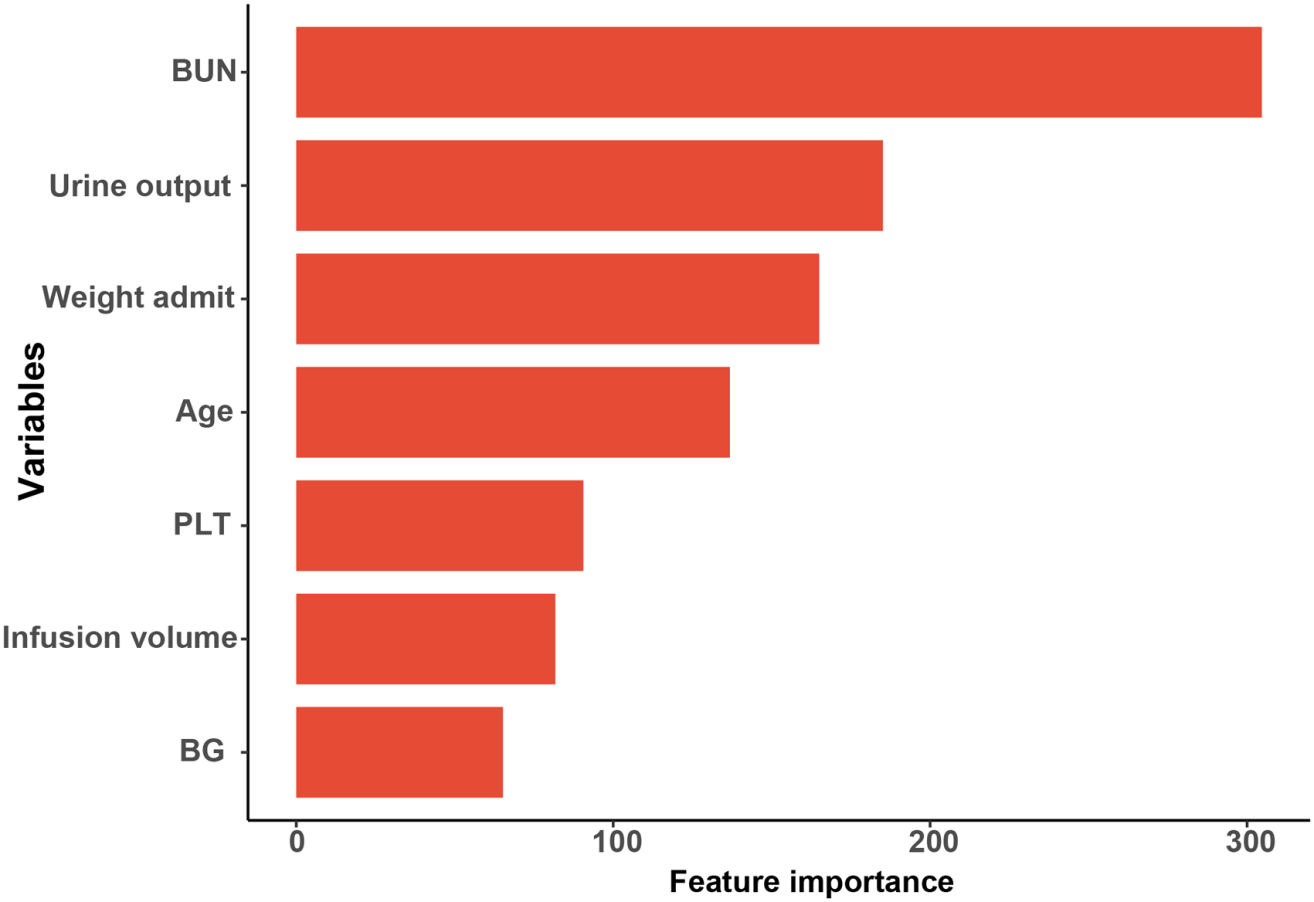
BUN, blood urea nitrogen; PLT, platelet count, BG, blood glucose.

Based on the XGBoost model with superior performance, 7 characteristics of interest were finally obtained in this study. *Feature importance* analysis was conducted to interpret the importance of the variables, which revealed that the top 5 contributing variables were blood urea nitrogen (BUN), urine output, weight, age, and platelet count (PLT), in that order (Figure 5 and Supplementary Table S3).

## 4. Discussion

Although the incidence of DKA-AKI was lower than in previous studies by Junzhe Chen (3) and Jean-Christophe Orban (8), it was comparable to the findings of our previous study in the MIMIC-III database (9). Thirty-eight candidate predictors were applied to train and validate 8 Ml models to predict the risk of DKA-AKI. The XGBoost model outperforms all other ML method in terms of discrimination and accuracy, with an AUC value of 0.835 in the training set and 0.800 in the validation set. BUN, urine output, weight, age, PLT, fluid volume, and glucose were the 7 variables that contributed to the XGBoost model orderly. The mortality rate among patients in the AKI group was found to be 10 times higher than that of the non-AKI group, with rates of 10.1 and 1.3%, respectively. Additionally, patients who suffered from AKI were more likely to require mechanical ventilation and spend longer periods of time in the ICU, leading to increased medical expenses. Accurately predicting the incidence of DKA-AKI can assist clinicians in identifying high-risk DKA-AKI patients in the ICU, and timely treatment and management can significantly improve the prognosis of these patients.

With the evolution of electronic medical and advent of the Big Data era, ML has already achieved remarkable achievements in the diagnosis and prognosis of diseases (17). For instance, ML methods have been maturely applied to develop predictive models for AKI in patients with sepsis, as well as in those undergoing cardiac and liver surgeries (13, 18–21).

BUN and urine output at baseline were included in our final model and were ranked higher in importance, so more attention should be given to patients with DKA who already had abnormal BUN and urine volume at ICU admission. Additionally, body weight was found to be a predictor for DKA-AKI in our study, with a median weight of 70.0 [IQR 60.3, 83.6] (Kg) in the AKI group compared to 78.5 [IQR 66.0, 94.0] (Kg) in the non-AKI group. Shi et al. (22) reported that overweight and obese patients were at a significantly higher risk of cardiac surgery-related AKI. However, since height is largely absent of ICU patients from the database, we were unable to calculate body mass index (BMI). Notably, increased intra-abdominal pressure in critically ill obese patients causes venous obstruction and insufficient blood flow to arterial organs, which may account for why obese patients are more likely to experience AKI (22–24). Age was also found to be significantly associated with AKI in our study, consistent with previous research (3, 8). The median age was higher in the AKI group (58 years [IQR 46, 68]) compared to the non-AKI group (43 years [IQR 30, 57]). Age-related structural and functional changes in the kidney include glomerulosclerosis, a decline in estimated glomerular filtration rate (eGFR), and an increase in glomerular capillary pressure. The kidney becomes more vulnerable to acute injury as it ages because its capacity to self-regulate declines (25). Long-term poor glycemic control, which is common in older patients with diabetes, can also lead to persistent kidney damage through inflammation, oxidative stress, and glycosylation (26). Both fluid therapy and low-dosage insulin are crucial treatments for DKA. Our study found that patients with AKI received more fluid infusions and experienced less urination compared to patients without AKI, leading to a higher cumulative fluid balance. Similar findings have been reported in earlier studies (27–29). Furthermore, Inkinen et al. (30) demonstrated that this phenomenon is linked to a lack of recovery from AKI. Excessive fluid intake may result in interstitial renal edema, which can raise renal perfusion pressure and impair kidney function. Ischemia–reperfusion (I/R) injury represents one of the crucial mechanisms of AKI. Following this, coagulation and inflammation are activated, and platelets are crucial to this process. In an animal model of AKI induced by I/R, Jansen et al. demonstrated that a significant proportion of activated platelets were present in the necrotic zone. Furthermore, the application of clopidogrel prior to modeling reduced tubular necrosis and preserved some renal function in mice (31). Also, an observational cohort study discovered that a significant connection between preoperative aspirin use and a reduced risk of AKI linked to cardiac surgery (32). Further research is necessary to determine whether antiplatelet medications can be utilized to prevent and treat other causes of AKI. BG was still included in the final XGBoost model and was negatively correlated with the outcome even though there was no statistically significant difference between the AKI (191.0 mg/dl [IQR 139.0, 258.0]) and the non-AKI groups (183 mg/dl [IQR 130.0, 251.0]) in terms of BG level. This could be due to the fact that individuals with kidney damage are more likely to experience hypoglycemia when taking insulin (33).

In contrast to previous clinical studies, we have successfully constructed a tool for assessing the risk of DAK-AKI based on several supervised ML algorithms. This model is constructed using a large real-world database and plays a crucial role in risk assessment and stratification of AKI, particularly in critically ill patients. To our knowledge, our study is the first to develop and validate a ML-based model of DKA-AKI, assisting clinicians in identifying high-risk individuals’ early and addressing associated risk factors promptly. Furthermore, our sample size of DKA patients is the largest compared to similar studies, ensuring more robust results. Encouragingly, the prediction models constructed in our study exhibit promising performance in both the training and validation sets.

Limitations of our present study should be noted. Firstly, our research was conducted as a single-center study on ICU patients, and we obtained data from the MIMIC-IV database. Further research will require data collection on patients from various nations or regions as well as general medical wards to enhance the generalizability of the findings.

Second, missing data were present in this study, and some variables with missing data of >20% were excluded. However, we made every effort to address the limitations of missing data by applying the KNN algorithm to the dataset for interpolation. Third, this is a retrospective study based on a public database the results have some limitations. Finally, in the absence of prior monitoring data in the MIMIC-IV database, it becomes challenging to assess if a patient with DKA has developed AKI. Hence, we cannot exclude this group of patients from the study, which may result in biased results.

## 5. Conclusion

Collectively, we extracted the data from the MIMIC-IV database to build ML-based model to predict the DKA-AKI. The results showed that BUN, urine output, weight, age, PLT, infusion volume, and glucose, in order of importance, were predictors of the occurrence of DKA-AKI. This model can identify high-risk patients at an early stage, assist clinical decision-making, and may improve the prognosis of DKA patients to some extent.

## Data availability statement

The raw data supporting the conclusions of this article will be made available by the authors, without undue reservation.

## Author contributions

TF, JW, and LL collected the data, analyzed the data, and drafted the manuscript. CZ supervised the project and reviewed the manuscript. TF, JK, WW, JW, and CZ conceived of the study, participated in its design and coordination, and helped to draft the manuscript. CZ was responsible for the whole project, designed the study, and supervised the study. All authors read and approved the final manuscript.

## Funding

This work was supported by Extreme Smart Analysis platform (https://www.xsmartanalysis.com/).

## Conflict of interest

The authors declare that the research was conducted in the absence of any commercial or financial relationships that could be construed as a potential conflict of interest.

## Publisher’s note

All claims expressed in this article are solely those of the authors and do not necessarily represent those of their affiliated organizations, or those of the publisher, the editors and the reviewers. Any product that may be evaluated in this article, or claim that may be made by its manufacturer, is not guaranteed or endorsed by the publisher.

## Footnotes

^1^ https://www.xsmartanalysis.com/

## Abbreviations

BG: Blood glucose
DM: Diabetes mellitus
AKI: Acute kidney injury
ICU: Intensive care unit
CKD: Chronic kidney disease
SCr: Serum creatinine
KDIGO: Kidney Disease Improving Global Outcomes
RR: Respiratory rate
BUN: Blood urea nitrogen
ML: Machine learning
MIMIC-IV: Marketplace for Medical Information in Intensive Care-IV
ICD: International Classification of Diseases
LASSO CV: Least absolute shrinkage and selection operator cross-validation
XGBoost: eXtreme Gradient Boosting
GNB: Gaussian Naïve Bayes
MLP: Multi-layer perceptron neural network
CNB: Complement Naive Bayes
SVM: Support vector machine
AUC: Area under the receiver operating characteristic curve
SD: Standard deviation
HLOS: Hospital length of stay
SBP: Systolic blood pressure
PLT: Platelet count
DCA: Decision curve analysis
BMI: Body mass index
eGFR: Estimated glomerular filtration rate
I/R: Ischemia–reperfusion
