# Supplementary material for: Predicting the risk factors of diabetic ketoacidosis-associated acute kidney injury: A machine learning approach using XGBoost
Source: Front Public Health. 2023 Apr 6;11:1087297. doi: 10.3389/fpubh.2023.1087297 (PMC10117643; doi:10.3389/fpubh.2023.1087297)
Supplement: Supplementary file 2 [file Data_Sheet_2.ZIP › Table S1.docx]

|  | **Variable** |
| --- | --- |
| Demographics |  |
|  | age, gender, ethnicity, height, weight |
| Vital signs |  |
|  | HR, RR, SBP, DBP |
| Characteristics of diabetes | |
|  | DM type, microangiopathy, macroangiopathy |
| Comorbidities |  |
|  | history of AMI, history of ACI, CHF, liver disease, preexisting-CKD, malignant cancer, hypertension, UTI, pneumonia |
| Laboratory test |  |
|  | WBC, lymphocyte, Hb, PLT, PO2, PCO2, PH, AG, bicarbonate, BUN, calcium, Scr, BG, phosphate, albumin, eGFR, HbA1C, CRP, urine ketone |
| Scoring systems |  |
|  | GCS, OASIS, SOFA, SAPSⅡ |
| Interventions |  |
|  | CRRT, MV, use of NaHCO3 |
| Prognosis |  |
|  | Hospital mortality, HLOS |

**Table S1** Variables extracted from the MIMIC-Ⅳ database.

**Abbreviations:** HR, heart rate; RR, respiratory rate; SBP, systolic blood pressure; DBP, diastolic blood pressure; DM, diabetes mellitus; AMI, acute myocardial infarction; ACI, acute cerebral infarction; CHF, congestive heart failure; CKD, chronic kidney diseases; UTI, urinary tract infection; WBC, white blood cell; Hb, hemoglobin; PLT, platelets count; PO2, partial pressure of oxygen; PCO2, Partial pressure of carbon dioxide; AG, anion gap; BUN, blood urea nitrogen; Scr, serum creatinine; BG, blood glucose (BG); eGFR, estimated glomerular filtration rate; HbA1C, hemoglobinA1c; CRP, C-reactive protein; GCS, Glasgow coma scale; OASIS, oxford acute severity of illness score; SOFA, sequential organ failure assessment; SAPS-Ⅱ, simplified acute physiology score II, SOFA sequential organ failure assessment; CRRT, continuous renal replacement therapy; MV, mechanical ventilation; HLOS, hospital length of stay.

**Formula:** AG=(Na^+^+K^+^)-(Cl^-^-HCO_3_^-^); Total osmotic pressure=2(Na^+^+K^+^)+ urea(mmol/l) +glucose (mmol/l).

**Other details:** Microangiopathy means patients with diabetic nephropathy, diabetic retinopathy, or diabetic peripheral neuropathy. Macroangiopathy means patients with coronary heart disease, cerebral atherosclerosis, or peripheral atherosclerosis.
