# Supplementary material for: Predicting the risk factors of diabetic ketoacidosis-associated acute kidney injury: A machine learning approach using XGBoost
Source: Front Public Health. 2023 Apr 6;11:1087297. doi: 10.3389/fpubh.2023.1087297 (PMC10117643; doi:10.3389/fpubh.2023.1087297)
Supplement: Supplementary file 2 [file Data_Sheet_2.ZIP › Table S2.docx]

| Excluded variable | Missing rate |
| --- | --- |
| Height | 66% |
| C-reactive protein | 94% |
| HbA1C | 61% |
| Albumin | 50% |
| Urine ketone | 69% |
| Lymphocytes | 64% |
| PH | 23% |
| PO2 | 23% |
| PCO2 | 23% |

**Table S2** Variables excluded due to missing rates greater than 20%.

**Abbreviations:** CRP, C-reactive protein; HbA1C, hemoglobinA1c; PO2, partial pressure of oxygen; PCO2, Partial pressure of carbon dioxide.
