# Supplementary material for: Predicting the risk factors of diabetic ketoacidosis-associated acute kidney injury: A machine learning approach using XGBoost
Source: Front Public Health. 2023 Apr 6;11:1087297. doi: 10.3389/fpubh.2023.1087297 (PMC10117643; doi:10.3389/fpubh.2023.1087297)
Supplement: Supplementary file 2 [file Data_Sheet_2.ZIP › Table S3.docx]

| Variable | Total Gain | Total Cover | Gain | Cover | Weight Importance |
| --- | --- | --- | --- | --- | --- |
| Bun | 304.666 | 58196.000 | 2.101 | 401.352 | 145 |
| Urine output | 185.104 | 75352.000 | 1.172 | 476.911 | 158 |
| Weight | 164.972 | 120343.000 | 0.685 | 499.349 | 241 |
| Age | 136.803 | 73734.000 | 0.819 | 441.521 | 167 |
| PLT | 90.575 | 67355.000 | 0.479 | 356.376 | 189 |
| Infusion volume | 81.743 | 60031.000 | 0.521 | 382.363 | 157 |
| Blood glucose | 65.210 | 73128.000 | 0.444 | 497.469 | 147 |

Table S3 Feature importance analysis.

**Abbreviations:** BUN, blood urea nitrogen; PLT, platelet count.
